# Humanization, Radiolabeling and Biodistribution Studies of an IgG_1_-Type Antibody Targeting Uncomplexed PSA for Theranostic Applications

**DOI:** 10.3390/ph14121251

**Published:** 2021-12-01

**Authors:** Joanna Strand, Kjell Sjöström, Urpo J. Lamminmaki, Oskar Vilhelmsson Timmermand, Sven-Erik Strand, Thuy A. Tran

**Affiliations:** 1 Department of Oncology, Department of Clinical Sciences, Lund University, 22243 Lund, Sweden; oskar.vilhelmsson_timmermand@med.lu.se (O.V.T.); sven-erik.strand@med.lu.se (S.-E.S.); 2 Innovagen AB, 22362 Lund, Sweden; kjell.sjostrom@innovagen.com; 3 Department of Biotechnology, University of Turku, 20500 Åbo, Finland; urplammi@utu.fi; 4 Department of Medical Radiation Physics, Department of Clinical Sciences, Lund University, 22243 Lund, Sweden; 5 Department of Oncology and Pathology, Karolinska Institutet, 17177 Stockholm, Sweden; 6 Department of Radiopharmacy, Karolinska University Hospital, 17177 Stockholm, Sweden

**Keywords:** prostate cancer, imaging, humanization, 5A10, fPSA, theranostics

## Abstract

Metastatic castration-resistant prostate cancer is today incurable. Conventional imaging methods have limited detection, affecting their ability to give an accurate outcome prognosis, and current therapies for metastatic prostate cancer are insufficient. This inevitably leads to patients relapsing with castration-resistant prostate cancer. Targeting prostate-specific antigens whose expression is closely linked to the activity in the androgen receptor pathway, and thus the pathogenesis of prostate cancer, is a possible way to increase specificity and reduce off-target effects. We have humanized and evaluated radioimmunoconjugates of a previously murine antibody, m5A10, targeting PSA intended for theranostics of hormone-refractory prostate cancer. The humanized antibody h5A10 was expressed in mammalian HEK293 cells transfected with the nucleotide sequences for the heavy and light chains of the antibody. Cell culture medium was filtered and purified by Protein G chromatography, and the buffer was changed to PBS pH 7.4 by dialysis. Murine and humanized 5A10 were conjugated with p-SCN-Bn-CHX-A”-DTPA. Surface plasmon resonance was used to characterize the binding to PSA of the immunoconjugates. Immunoconjugates were labeled with either indium-111 or lutetium-177. Biodistribution studies of murine and humanized 5A10 were performed in mice with LNCaP xenografts. 5A10 was successfully humanized, and in vivo targeting showed specific binding in xenografts. The results thus give an excellent platform for further theranostic development of humanized 5A10 for clinical applications.

## 1. Introduction

Prostate cancer (PCa) is one of the major causes of death among men in the western world. First-line hormonal therapy of metastatic PCa is initially a very effective treatment for metastatic PCa. Androgen ablation, with e.g., anti-androgens and GnRH-agonists/antagonists, inhibits the androgen receptor (AR) pathway central to survival, proliferation, and apoptosis of healthy and malignant prostate cells [1,2]. Over time, however, reactivation of the AR signaling axis occurs, which eventually leads to castration-resistant prostate cancer (CRPC), for which there is no curative treatment. Despite an expanding arsenal of targeted agents to treat and monitor metastatic PCa, the disease remains incurable. Conventional imaging modalities available in the clinic, such as computed tomography (CT), magnetic resonance tomography (MR) and bone scan are of limited use for the detection of metastatic PCa. This contributes to a lack of clinical insight into the biomolecular effects, i.e., androgen axis activity, and into the efficacy of existing therapies. New targeted radionuclide therapy and imaging modalities aiming directly at AR pathway activity are thus a potential promise in metastatic PCa and CRPC patients.

Recently the introduction of imaging of prostate-specific membrane antigen (PSMA) represented the first clinical breakthrough in PET imaging [3] focusing on targets downstream of the androgen receptor (AR) pathway [4]. Furthermore, both lutetium-177-labeled PSMA targeted antibodies (J591) and small molecules have shown promising anti-tumor effects. However, PSMA is fairly non-tissue-specific with uptake in salivary glands, kidneys and nerve endings. Prostate-specific antigen (PSA), on the other hand, is an antigen expressed almost exclusively by healthy and malignant prostate epithelial cells [5]. Several pathological processes, e.g., malignancy and inflammation, cause a retrograde release of PSA into the blood. Here, however, serine protease inhibitors, such as alpha 1-antichymotrypsin, bind to and block the catalytic cleft, forming a stable complex with PSA [6]. PSA expression is directly governed by the AR pathway and thus follows the criteria above as a clinically relevant target for new therapy and imaging approaches.

Recently, our group developed several radioimmunoconjugates based on the monoclonal murine IgG_1_, m5A10. This IgG binds specifically to an epitope on the catalytic cleft of uncomplexed, free-PSA (fPSA), and has shown promising results of localization, verified with PET-imaging using zirconium-89 (^89^Zr) and uptake studies using indium-111 (^111^In) labeled m5A10 [7,8]. PET-imaging also demonstrated that different PSA expression levels on tumors of castration-resistant prostate cancer can be quantitatively measured using ^89^Zr-m5A10 [8].

Given these promising results using the murine 5A10, we here report on the humanization of the antibody, to reduce the immunogenicity of the 5A10 antibody in man, and thus allow it to be used in the clinic. The internalization and retention of antibodies binding to secreted antigens, e.g., PSA, are dependent on binding to the neonatal Fc-receptor (FcRn) [9]. When complexed with PSA, FcRn mediates a permanent cellular internalization of IgG_1_-type antibodies, as 5A10, resulting in cellular accumulation of therapeutic radionuclides when 5A10 is labeled with a therapeutic radionuclide as ^177^Lu [9].

This study aimed to compare the targeting properties of radioimmunoconjugates of humanized 5A10 with the murine predecessor, and further explore the theranostic possibility of h5A10. We assessed the in vivo kinetics when labeled with ^111^In intended for diagnostics. Additionally, we studied the biodistribution profile of ^177^Lu-labeled humanized 5A10 (h5A10). By changing the diagnostic radionuclide to the β-emitter ^177^Lu, we are able to use the same targeting agent for therapy. We present here the biokinetics, being the base for further therapeutic studies of this new radioimmunoconjugate. Furthermore, a basic developability assessment study regarding h5A10 has been performed. Collectively, the results will provide radioconjugates with favorable characteristics for further radioimmunotherapy studies.

## 2. Result

### 2.1. Humanization and Characterizations

**Figure 1:**
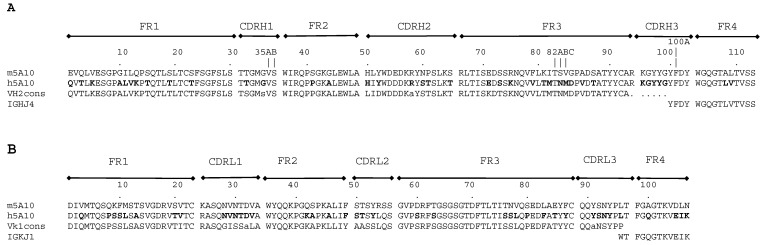
Sequence alignment of humanized 5A10, parental murine antibody m5A10, and the template sequences used for humanization. (Panel **A**) shows the sequences of the heavy and (Panel **B**) those of the light chain variable domains. In the case of the heavy chain, the template sequences comprise the consensus sequence of V_H_2-family and IGHJ4-gene encoded segment and in the case of the light chain, the consensus sequence of V_k_1-family and IGKJ1-gene encoded segment, respectively. The positions where m5A10 and the template sequences differ are bolded in h5A10. The framework (FR) and the complementarity determining regions (CDRs) of the variable domains are indicated. CDR definitions and numbering are according to [10]. The characters with small letters in the consensus sequences stand for: s = “small residue” (A,C,D,G,N,P,S,T,V) and a = “aromatic residue” (F,H,W,Y).

**Figure 2:**
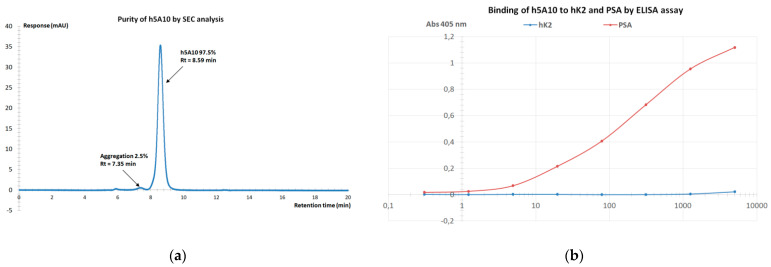
(**a**) The purity and possible aggregation of h5A10 analyzed by size exclusion chromatography (SEC) analysis, at 37 °C (**b**) The possible cross−reactivity of h5A10 towards hK2 was analyzed by ELISA.

The amino acid sequences of heavy or light chain variable domains of h5A10 are displayed in Figure 1. For further functional characterization, h5A10 was produced in IgG_1_ format in HEK293 cells and purified, up to ~97.5% purity with an overall yield of 15 mg. The h5A10 IgG_1_ showed high binding activity towards PSA and practically no cross-reactivity to hK2, another human kallikrein with very high sequence similarity to PSA (see Figure 2b).

### 2.2. Binding Kinetics and Affinity of Immunoconjugates

The binding kinetics of the non-conjugated and diethylenetriaminepentaacetic acid(DTPA)-conjugated showed subnanomolar affinities (K_D_) to the fPSA antigen (Table 1). The affinities were in the range of 10–20 pM. DTPA conjugation affects somewhat the affinity of the m5A10. There was no unspecific binding to the control hK2 antigen, which is PSA homologous (data not shown).

### 2.3. Radiolabeling and Stability Results

**Figure 3:**
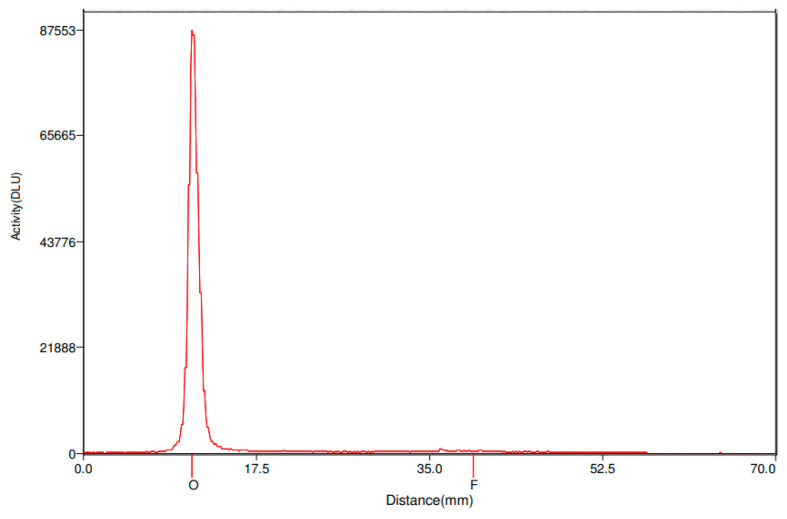
Radiochemical yield and purity assessed by iTLC. Here is a typical chromatogram of ^177^Lu-DTPA-h5A10. Distribution of radioactivity along the iTLC was visualized and quantified using Cyclone Storage Phosphor System. The radiolabelled product remains at the origin while any free radionuclide migrates with the front.

The conjugates were efficiently labeled with ^111^In and the labeling yields were 70–80%. Radiochemical purity, analyzed by instant thin layer chromatography (iTLC), after purification was >95%. See Figure 3 for a typical iTLC chromatogram. The shelf-life of the products was stable up to 14 days post end-of-synthesis (EOS), with >95% of radioactivity remaining attached to the conjugate, in PBS, as analyzed by iTLC. Specific activities were in the range of 0.28–0.40 MBq/µg. Radiolabeling of the immunoconjugate DTPA-h5A10 with ^177^Lu was successful with a radiochemical yield of 92 ± 1% (*n* = 3) and a radiochemical purity of 99 ± 0.5% (*n* = 3). Moreover, radiolabeled h5A10 demonstrated high stability after the challenge with 500-fold molar excess of ethylenediaminetetraacetic acid (EDTA). The majority of ^177^Lu (86 ± 1%) was still bound to h5A10 at 48 h post-EDTA incubation.

### 2.4. Evaluation of h5A10 Versus m5A10 for Diagnostic SPECT Imaging

**Figure 4:**
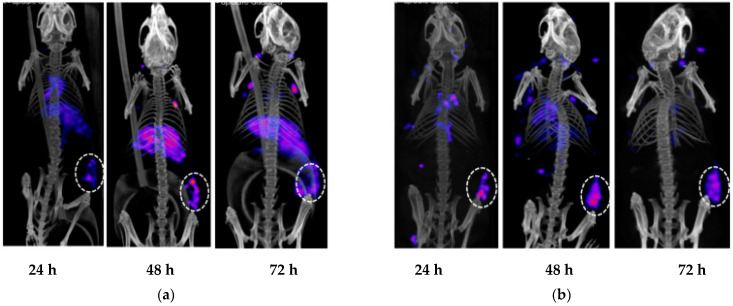
Small animal SPECT/CT imaging of LnCAP xenografts with (**a**) ^111^In-DTPA-m5A10 and (**b**) ^111^In-DTPA-h5A10. The in vivo targeting of ^111^In-labeled m5A10-DTPA or h5A10-DTPA was verified in LnCAP-bearing xenografts. Tumors are well visualized on the right flank, as indicated in rings. The images are scalled to the same intensity. Higher radioactivity in the liver is observed for the murine compared to the humanized 5A10. Region-of-interest (ROI) analysis at the SPECT images at 24 h showed a liver accumulation of 7 % of injected activity for m5A10 as compared to 5 % for h5A10. These values are within the uncertainty limits.

To confirm that 5A10 retained its in vivo targeting capability to fPSA after humanization, single-photon emission computed tomography, SPECT/CT, imaging of ^111^In-DTPA-h5A10 and ^111^In-DTPA-m5A10 was performed. Figure 4 shows representative SPECT/CT images of LNCaP xenografts measured at 24, 48, 72, h post-injection of ^111^In-DTPA-m5A10 (Figure 4a) and ^111^In-DTPA-h5A10 (Figure 4b). The tumors were clearly visualized already after 24 h, but the contrast was improved over time, particularly for the h5A10. Higher radioactivity in the liver was also observed (Figure 4) for the murine compared to the humanized 5A10. Region-of-interest (ROI) analysis at the SPECT images at 24 h showed a liver accumulation of 7 % of injected activity for m5A10 as compared to 5 % for h5A10. These values are within the uncertainty limits.

### 2.5. Biodistribution Studies of ^177^Lu-h5A10 and Specificity

**Figure 5:**
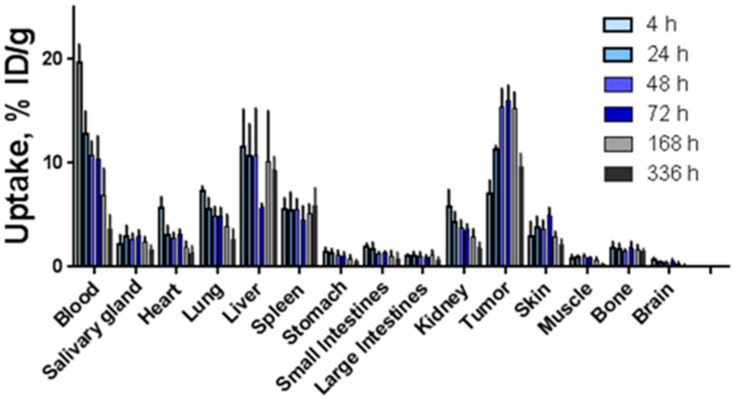
Biodistribution of ^177^Lu-DTPA-h5A10 in LnCAP xenografts over time up to 336 h post-injection. The uptake is expressed as % injected dose per gram tissue (%ID/g), and presented as an average ± SD (*n = 4*).

**Figure 6:**
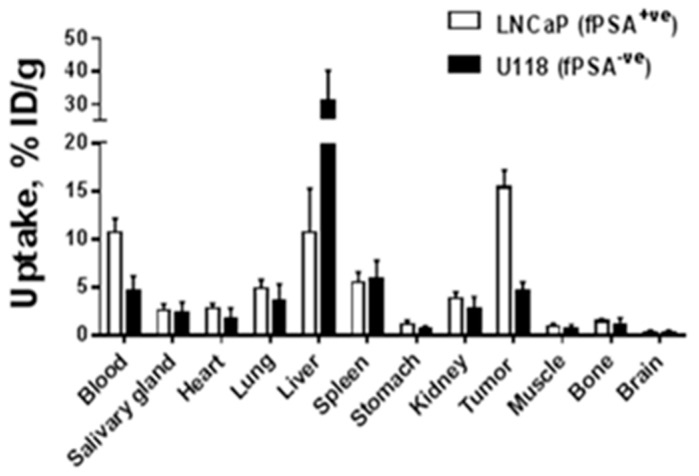
Comparative biodistribution of ^177^Lu-h5A10 in NMRI mice bearing subcutaneous LNCaP (fPSA-expressing) and U118-glioblastoma (non-fPSA-secreting) xenografts 48 h p.i. The uptake is expressed as % injected dose per gram tissue (%ID/g), and presented as an average ± SD (*n = 4*). Note the high specificity for ^177^Lu-h5A10, *p* < 0.001.

The biodistribution of ^177^Lu-h5A10 at 4, 24, 48, 72, 168 and 336 h p.i. in BALB/c-nu/nu mice bearing fPSA-secreting-LNCaP xenografts is displayed in Figure 5. In brief, at early time points the concentration of radioactivity in the blood was the highest among all studied tissues (19.64 ± 1.76%IA/g and 12.75 ± 2.23 %IA/gat 4 and 24 h p.i.). Forty-eight hours p.i. the blood-borne radioactivity decreased by approx. 50% (10.75 ± 1.36 %IA/g) and the tumor-associated radioactivity peaked at (15.39 ± 1.76 %IA/g). By this time the tumor-to-blood radioactivity uptake ratio was 1.5 ± 0.2 (Table 2) and continued to increase over time (3.0 ± 1.3 at 336 h p.i.). ^177^Lu-h5A10 demonstrated high retention in the tumor over time (15.4 ± 1.8, 15.0 ± 2.3 and 15.2 ± 1.6 %IA/g at 48, 72 and 168 h p.i.). Besides the tumor, the liver was the only normal organ with a prominent accumulation of radioactivity (Figure 5 and Table 2). To test for specific tumor accumulation of ^177^Lu-CHX-A’’-DTPA-h5A10 in vivo, uptake of the radioimmunoconjugate in fPSA-positive (LNCaP-prostate cancer) and fPSA-negative (U118-glioblastoma tumors) tumors was measured at 48 h p.i. The results of the in vivo specificity test are presented in Figure 6. The uptake in fPSA-negative U118 glioblastoma xenografts was significantly lower (*p* < 0.001) than the uptake in fPSA-positive LNCaP xenografts (Figure 6), confirming the specificity of h5A10 for fPSA.

## 3. Discussion

Humanization of the murine IgG_1_ antibody 5A10 was performed in this study to reduce/eliminate the immunogenicity prior to future use in humans in the clinic. In addition to grafting the CDR sequences from murine 5A10 to a suitable human framework, the development of the h5A10 involved the introduction of human-to-mouse and some mouse-to-human backmutations in the framework regions of the CDRs. After humanization, we performed measurements of the overall affinity (K_D_), radiolabeled the mAb and preclinically evaluated the targeting properties of h5A10 IgG in PSA-expressing LnCAP xenografts, with a head-to-head comparison with the murine predecessor.

We showed that the humanized 5A10 antibody could efficiently target the fPSA in the same manner as the murine 5A10. Although the SPR data indicated that the conjugation process might affect somewhat the binding affinity to the target for both conjugates, the K_D_ values are still on the subnanomolar levels and were in line with other repeated measurements [11]. The murine predecessor m5A10, labeled with ^89^Zr [8] has been previously evaluated using PET. Similar to these previous PET results, ^111^In-m5A10 and ^111^In-h5A10 are also efficient in the detection of PSA-expressing tumors in mice in preclinical SPECT imaging. Furthermore, we have recently confirmed PSA imaging of h5A10 mAb using ^89^Zr-labeled h5A10 in both LnCaP mouse models and in non-human primates [9], confirming the retained activity after the humanization process.

The tumor accumulation was highest at 48 h post-injection for ^177^Lu-labeled h5A10 (15.4 ± 1.8 %IA/g). Interestingly the accumulation in the tumor retained a stable retention up to 7 days post-injection (15.2 ± 1.6 %IA/g). In comparison with a previous study for ^111^In-labeled murine 5A10 the highest tumor uptake was measured as early as 24 h post-injection followed by a tumor clearance at later time points [7]. In the present study the highest uptake for normal organs was observed in the liver, 11.5 ± 3.66–9.32 ± 1.29 %IA/g between 4 and 336 h p.i. High hepatic uptake for m5A10 has been observed earlier [7,8]. Our group has previously found that the neonatal Fc receptor mediates a permanent internalization of IgG_1_-type antibodies complexed with secreted antigens (such as 5A10). One might speculate that h5A10 is trapped in the liver, as hepatocytes express FcRn, but this would require a more thorough investigation in the future to explore the mechanism of live uptake of h5A10. The results are in good agreemeent with previously published data for both the murine and humanized version of 5A10, with retained specificity for fPSA and low uptake in normal organs [8,9,11]. Significantly lower tumor uptake was seen in non-PSA expressing tumors (Figure 6), suggested that this uptake was PSA-specific.

Further, the sequence homology between the h5A10 target, PSA, and other human proteins has been investigated. Finally, the cross-reactivity towards the most similar protein, human kallikrein-2 (hK2) was evaluated and no cross-reactivity to hK2 was found. In addition, the h5A10 seems to be very stable over time, showing no sign of aggregation. However, small molecular changes during six years’ storage at +4 °C were identified by physicochemical analytical methods, such as increased Mw of the heavy chain and increased charge heterogeneity (data not shown). No molecular changes could be detected by the selected physicochemical analytical methods during the limited forced degradation study.

Basic local alignment search tool (BLAST) analysis revealed that the most similar human protein to PSA by sequence is the human kallikrein-2. When aligned, almost 80% of the amino acids are identical. There are seven stretches with 10 identical amino acids or more (100% homology), which could very well lead to some cross-reaction for h5A10 towards hK2. To dispel any suspicions, an ELISA analysis was performed, and the result clearly shows that there is no cross-reaction of h5A10 to hK2. There is no other obvious protein candidate with which h5A10 may cross-react. Improved performance of humanized mAbs may be attributed to improved avidity and attenuated humoral response. For example, the mAb J591 [12], which targets an extracellular epitope of human PSMA with a very high affinity (*K_D_* = 1.83 ± 1.21 nM) was first developed of murine origin. J591 is mainly used for therapeutic applications of disseminated prostate cancer using ^177^Lu [13,14,15]. In comparison with J591, ^177^Lu-h5A10 demonstrated a comparable tumor accumulation in LNCaP-xenografts. For example, the specific uptake (%IA/g) of tumor was 15.4 ± 1.8 for ^177^Lu-h5A10 at 48 h p.i. vs. 15.95 ± 2.13 for ^177^Lu-radiolabeled J591 [14,16].

In summary, we present in this work a stable, highly specific fPSA-targeting humanized 5A10 monoclonal antibody. The results in the present study give an excellent platform for further theranostic development of humanized 5A10 for clinical applications.

## 4. Materials and Methods

### 4.1. Humanization of m5A10

The variable domains of m5A10 were humanized with CDR (complementarity determining region) grafting approach [17], i.e., the CDRs of m5A10 were introduced in a variable domain framework encoded by human immunoglobulin genes. In order to identify suitable human frameworks, the light and heavy chain variable domain sequences of m5A10 were compared to the amino acid sequences of the human immunoglobulin germline V- and J-genes available in NCBI using the ClustalW program. Consensus sequences were deduced for the V-gene families that showed the highest similarity to m5A10 using a web-server-based computational tool (http://coot.embl.de/Alignment/consensus.htm, accessed on 5 May 2021). These consensus sequences were used as templates for the main part of V_H_ and V_L_-segments of h5A10 (FR1, 2 and 3 regions; Figure 1). The J-gene encoded segments showing highest similarities to the corresponding m5A10 sequences were used as templates for the C-terminal parts of the V-domains (FR4 region; Figure 1). CDR loop sequences of m5A10 were introduced into these human V_L_ and V_H_ framework sequences. A homology model was generated for the 3D structure of m5A10 using the automatic Web Antibody Modelling server (WAM; http://antibody.bath.ac.uk/index.html, accessed on 5 May 2021). The homology model, in concert with the sequence alignment and published information [18], was used to assess the role of individual variable domain residues in terms of the binding and folding properties of h5A10. Based on this analysis, certain non-CDR loop residues were retained as in m5A10.

### 4.2. Expression, Purification, SEC and ELISA Analysis of the Humanized 5A10 IgG_1_

The murine 5A10, denoted as m5A10, was provided by the University of Turku (Finland) and was prepared as previously described [8], while the humanized 5A10, denoted as h5A10, for this study was expressed and purified as follows: HEK293 cells were expanded into a 2L suspension culture in FreeStyle 293 Expression Medium (Life Technologies, Carlsbad, CA, USA). The cell density on the day of transfection was 1 × 10^6^ cells/mL. The nucleotide sequences encoding the component heavy or light chains (see the sequence in Figure 1) were codon-optimized for expression in mammalian cells, synthesized and cloned to IgG expression vectors. The plasmid DNA (expression vector) containing the nucleotide sequences for the heavy and light chains was then mixed with the transfection agent and incubated for 10 min in RT. The DNA amount for the light chain, p5A10VlhDhK (4300 bp) was 0.35 mg and the heavy chain, p5A10VHhDhlgG1 (4900 bp) was 0.60 mg. The DNA-transfection agent-mix was slowly added to cell culture as the flask was slowly swirled. The transfected cell culture was then incubated at 37 °C, 8% CO_2_ on an orbital shaker platform rotating at approx. 135 rpm, for seven days. Culture medium was harvested by centrifugation and filtered through 5 µm, 0.6 µm and 0.22 µm filter systems. Antibodies were purified by Protein G chromatography and the buffer was changed to PBS pH 7.4 by dialysis; subsequently, the antibodies were concentrated by ultrafiltration.

Purity determination was analyzed by Size-Exclusion High-Performance Liquid Chromatography with a TSK-gel^®^ SuperSWmAb HTP column, (4 µm, 4.6 × 150 mm, Tosoh Bioscience, Griesheim, Germany) which separates molecules according to their molecular weight. Eluting peaks were detected using a UV detector. The analysis was run at a flowrate of 0.35 mL/min at and the buffer was PBS, pH 7.2. The integrated surface area was automatically calculated by the software Chromeleon 7.2 (Waltham, MA, USA).

Cross-reactivity and binding of PSA was determined by ELISA: Wells in a microtiter plate (Nunc Maxisorp, Sigma Aldrich, Saint Louis, MO, USA) were coated with PSA (Merck, Kenilworth, NJ, USA) or hK2 (Innovagen AB, Lund, Sweden) (100 µL at 1 µg/mL) overnight. After washing and blocking, a dilution series in duplicate, starting with 5 µg/mL and with four times dilution steps, was added to the wells and incubated in RT for one hour. After the plate was washed, a secondary antibody, Goat Anti-Human-AP, conjugated to alkaline phosphatase (Sigma-Aldrich, St. Louis, MO, USA) was diluted 10000× and 100 µL was added to each well. After incubation for one hour in RT, the plate was washed and 100 µL of pNPP solution was added to the wells (1 mg/mL, Sigma-Aldrich). Absorbance at 405 nm was measured with a Multiscan EX spectrophotometer (ThermoFisher, Waltham, MA, USA).

### 4.3. Conjugation and Binding Affinities by Surface Plasmon Resonance

All conjugation and labeling buffers were incubated with Chelex (Sigma Aldrich, St. Louis, MO, USA) overnight to reduce metal impurities that might interfere with radiolabeling efficiency. Murine 5A10 and humanized 5A10 was conjugated with p-SCN-Bn-CHX-A”-DTPA, from Macrocyclics (Dallas, TX, USA); at a 3:1 chelator to antibody molar ratio following the procedure below. Buffer exchange was conducted on PBS solutions of the antibodies, h5A10 and m5A10 (Innovagen AB). This was done by first equilibrating NAP-5 columns (GE Healthcare, Uppsala, Sweden) with 0.07 M sodium borate buffer pH 9.2 (Sigma Aldrich) and then eluting the antibodies on the columns using the same buffer. The reaction vial was incubated at 38 °C overnight. The antibody conjugates were separated from free chelate by size-exclusion chromatography using a NAP-5 column, preequilibrated with 20 mL 0.2 M pH 5.5 ammonium acetate buffer (Sigma Aldrich), through elution with 1 mL of the same ammonium acetate buffer. Aliquots were stored at –20 °C before labeling. The average number of DTPA chelators per antibody was determined as previously [11], and inspired by Mears et al. [19]. In this assay, the conjugates were mixed with ^111^InCl_3_/^nat^InCl_3_ solution consisting of known concentrations of non-radioactive (Sigma-Aldrich) and radioactive (^111^In) indium. The radioactive yield, together with the assumption of a 1:1 interaction between indium atoms and chelators, was utilized for calculating the number chelates attached to each antibody. The average number of DTPA chelators was 1.4 and 9.4 per antibody for 3:1 ratio and 12:1 ratio respectively.

Surface plasmon resonance (SPR) was used to study the interaction between the antigen and the antibody conjugate, using a Biacore 2000 system (GE Healthcare). A solution containing 3.0 µg/mL of the antigen PSA was immobilized on a CM4 research grade chip (GE Healthcare) via covalent amine coupling, with a flow rate of 5 µL/mL. The surface of the chip was activated using the contents of an amine coupling kit (GE Healthcare) containing 1-ethyl-3-(3-dimethylaminopropyl)carbodiimide hydrochloride (EDC) and N-hydroxysuccinimide (NHS). An immobilization of 900–1000 response units (RU) was achieved for PSA. For measurement, four different concentrations (100, 50, 25, 12.5 and 10 nM) of the unconjugated antibodies, and the different conjugated antibodies, all diluted in HSP-buffer, were flown over the immobilized channels fc2–4 with a flow rate of 30 μL/min. The association phase of the immunoconjugate was followed for 4–5 min and the dissociation phase was extended up to 480 min. The results were analyzed by subtracting the signal in the blank from that of the other flow cells, then fitting two separate bi-exponential fits to the binding and the dissociation curve respectively. From these the affinity of each immunoconjugate could be calculated.

### 4.4. Labeling Chemistry

For in vivo experiments variants of the mAb 5A10 were labeled with ^111^In or ^177^Lu. In brief, for SPECT imaging using ^111^In, approximately 50 µg of either m5A10 or h5A10 (conjugated to CHX-A”-DTPA in a 3:1 chelator-to-5A10 ratio) were mixed with a solution of approximately 10–15 MBq ^111^InCl_3_ (Mallinkrodt, Railroad Avenue, Hobart, NY, USA) in the presence of 0.2 M ammonium acetate buffer, pH 5.5. The mixture was vortexed and incubated at 38 °C for 1 h. The labeling efficiency of all radiolabeled conjugates was monitored using instant thin-layer chromatography strips, eluted with 0.2 M citric acid (Sigma Aldrich) and quantified with a Phosphor Imager system with the software Optiquant (Perkin Elmer, Wellesley, MA, USA). All radiolabeled conjugates were purified from non-bound ^111^InCl_3_ on a size-exclusion NAP-5 column (Thermo Fischer Scientific) equilibrated with PBS (Hyclone). For labeling with ^177^Lu (Curium, Stockholm, Sweden), 30–50 μg of the conjugate (12:1 chelator to h5A10 ratio) was mixed with ^177^LuCl_3_ (15–25 MBq) in ammonium acetate buffer as above. The radioconjugate was vortexed and incubated at 38 °C. The radiolabeled conjugate was purified using AMICON Ultra-0.5-centrifugal filter devices with a MWCO 30,000 Da (Millipore, Burlington, MA, USA). The radioconjugate labeling yield and purity was determined ITLC strips (150–771 DARK GREEN Tec-Control Chromatography strips, Biodex Medical Systems, (Ramsey Road Shirley, NY, USA) eluted with 0.2 M citric acid and measured using the Cyclone Storage Phosphor System (PerkinElmer, Waltham, MA, USA). In this system, the radiolabelled product remained at the origin, while free ^111^In or ^177^Lu migrated with the front.

### 4.5. Animal Studies

Cell lines: PSA-expressing LNCaP cell line and U118 glio-blastoma cells were purchased from American Type Culture Collection. Cells were cultured in RPMI 1640 medium (Thermo Scientific) with 10% fetal bovine serum (Thermo Scientific), penicillin (100 IU/mL), and streptomycin (100 µg/mL) (Thermo Scientific). The cells were maintained at 37 °C in a humidified incubator at 5% CO_2_ and detached/passaged using trypsin-EDTA solution (Thermo Scientific).

All animal experiments (biodistribution and microSPECT/CT imaging) were performed in accordance with national legislation on laboratory animal protection and permitted by the Local Ethics Committee for Animal Research at Lund University. LNCaP cells (10^7^ cells per mouse) or U118 glio-blastoma cells (5 × 10^6^ cells/mouse) (fPSA negative) were implanted subcutaneously (s.c.), in a 1:1 mixture of matrigel (Corning, Corning, New York, USA) and RPMI 1640 medium, on the right hind leg of NMRI nude mice (Charles River, Wilmington, Massachusetts, USA). The xenografts were allowed to grow 3–5 weeks before the experiment.

For small animal SPECT imaging, NMRI-nu mice with s.c. LNCaP xenografts (*n* = 4 per group) were given a tail-vein injection, ~8–12 MBq, 50 µg mAb, of either ^111^In-DTPA-m5A10 or ^111^In-DTPA-h5A10 and imaged with nanoSPECT/CT (Bioscan, Budapest, Hungary), under isoflurane anesthesia (Abbott Scandinavia, Solna, Sweden) at 24, 48, 72 h. SPECT images were obtained using the NSP-106 multi-pinhole mouse collimator for approximately 50 min with energy windows of 20% centered over the 171.3 keV and 245.4 keV energy peaks. SPECT data were reconstructed using HiSPECT software at standard settings (SciVis, Goettingen, Germany). The SPECT/CT images were analyzed using VivoQuant 1.22 software (inviCRo, Boston, MA, USA).

Biodistribution studies of ^177^Lu-h5A10: Sixteen LNCAP-bearing NMRI-nu mice were randomized into six groups (four mice/group). Four groups were intravenously injected with 100 μL of ^177^Lu-h5A10 (20 μg in 2% BSA-PBS, 130 kBq, 0.13 mM EDTA) in the tail vein and euthanized using an overdose of anesthesia (20 μL of Ketalar-Rompun per gram body weight: Ketalar (50 mg/mL; Pfizer, New-York, NY, USA), 10 mg/mL; Rompun, (20 mg/mL; Bayer, r, Leverkusen, Germany) followed by heart puncture and exsanguination with a syringe at 4, 24, 48 and 72 h p.i. The remaining two groups were injected with 260 kBq/mouse of ^177^Lu-h5A10 (20 μg in 2% BSA-PBS, 130 kBq, 0.13 mM EDTA) and euthanized at 168 and 336 h p.i.

Blood, salivary gland, heart, lung, liver, spleen, stomach, small intestine, large intestine, kidney, tumor, skin, muscle, bone, brain and the remaining carcass were collected and weighed, and their radioactivity concentration in the respective tissue was measured in a NaI(Tl) automated well counter (PerkinElmer). To evaluate the specificity of radiolabeled h5A10, four U118-bearing mice were i.v. injected with ^177^Lu -h5A10 and mice were sacrificed at 48 h p.i. as described above. 

Data analysis was performed using GraphPad Prism version 4 (GraphPad Software Inc., San Diego, CA, USA) and Excel 2010 (Microsoft, Redmond, Washington, USA). Data are given as the mean ± SD or mean ± SEM and analyzed using T-test. All *p*-values under 0.05 are considered statistically significant, unless otherwise stated.

## Figures and Tables

**Table 1:** Binding kinetics and affinities by surface plasmon resonance (SPR).

Conjugate	K_off_ (10^−6^s^−1^)	K_on_ (10^6^ M^−1^s^−1^)	K_D_ (10^−12^ M)
m5A10	5.4	0.25	22
h5A10	4.8	0.62	7.7
m5A10-DTPA	6.3	0.27	23
h5A10-DTPA	4.8	0.39	12

^1^ Binding affinity, K_D_ = K_off_/K_on._

**Table 2:** Tumor-to-tissue ratios ± SD (*n = 4 per time point*) for ^177^Lu-h5A10.

Organs	4 h	24 h	48 h	72 h	168 h	336 h
Blood	0.4 ± 0.1	0.2 ± 0.0	1.5 ± 0.2	1.5 ± 1.0	2.4 ± 0.8	3.0 ± 1.3
Salivary gland	3.6 ± 0.17	0.3 ± 0.0	5.3 ± 0.8	5.2 ± 1.0	6.9 ± 2.1	6.1 ± 2.0
Heart	1.3 ± 0.4	1.6 ± 0.5	5.4 ± 1.0	4.8 ± 0.5	8.8 ± 2.7	8.5 ± 4.4
Lung	1.0 ± 0.2	1.5 ± 0.2	2.9 ± 0.4	3.1 ± 0.5	4.1 ± 1.1	4.1 ± 1.8
Liver	0.7 ± 0.2	0.8 ± 0.0	1.9 ± 0.9	2.6 ± 0.3	1.8 ± 1.0	1.0 ± 0.2
Spleen	1.3 ± 0.4	0.6 ± 0.4	2.9 ± 0.9	3.4 ± 0.8	3.1 ± 0.9	1.8 ± 0.7
Stomach	5.3 ± 2.5	0.8 ± 0.1	13.0 ± 3.7	15.3 ± 4.4	21.5 ± 9.2	19.6 ± 7.5
Small intestines	3.9 ± 1.3	3.3 ± 0.4	11.7 ± 0.2	11.1 ± 2.6	18.8 ± 11.8	19.6 ± 14.1
Large intestines	6.9 ± 2.3	2.8 ± 0.6	13.5 ± 4.2	17.4 ± 2.6	16.9 ± 7.3	18.0 ± 8.1
Kidneys	1.3 ± 0.5	4.1 ± 1.1	3.8 ± 0.5	4.2 ± 0.6	5.4 ± 1.2	5.6 ± 1.9
Skin	2.9± 1.5	0.5 ± 0.2	3.9 ± 0.5	3.1 ± 0.8	5.2 ± 0.4	4.9 ± 1.7
Muscle	9.9 ± 5.6	1.1 ± 1.1	14.6 ± 1.5	16.9 ± 2.8	25.5 ± 10.0	35.2 ± 13.2
Bone	4.7 ± 1.7	4.7 ± 1.0	9.9 ± 1.7	8.8 ± 2.5	9.6 ± 2.9	6.4 ± 1.2
Brain	12.5 ± 7.8	2.8 ± 0.8	38.4 ± 8.4	33.9 ± 15.9	74.1 ± 66.6	67.1 ± 44.7

## Data Availability

Data are available upon request to corresponding authors.
